# A perspective on persistent toxicants in veterans and amyotrophic lateral sclerosis: identifying exposures determining higher ALS risk

**DOI:** 10.1007/s00415-021-10928-5

**Published:** 2022-01-01

**Authors:** Diane B. Re, Beizhan Yan, Lilian Calderón-Garcidueñas, Angeline S. Andrew, Maeve Tischbein, Elijah W. Stommel

**Affiliations:** 1 Department of Environmental Health Science, Center for Motor Neuron Biology and Disease, Columbia University, New York, NY USA; 2 Department of Geochemistry, Lamont-Doherty Earth Observatory of Columbia University, Palisades, NY USA; 3 Department Biomedical Sciences, College of Health, University of Montana, Missoula, MT USA; 4 Universidad del Valle de México, Mexico City, Mexico; 5 Department of Neurology, Geisel School of Medicine at Dartmouth, Dartmouth-Hitchcock Medical Center, Lebanon, NH USA

**Keywords:** Amyotrophic lateral sclerosis, Veterans, Etiology, Neurotoxicant, Persistent, Exposure

## Abstract

Multiple studies indicate that United States veterans have an increased risk of developing amyotrophic lateral sclerosis (ALS) compared to civilians. However, the responsible etiological factors are unknown. In the general population, specific occupational (e.g. truck drivers, airline pilots) and environmental exposures (e.g. metals, pesticides) are associated with an increased ALS risk. As such, the increased prevalence of ALS in veterans strongly suggests that there are exposures experienced by military personnel that are disproportionate to civilians. During service, veterans may encounter numerous neurotoxic exposures (e.g. burn pits, engine exhaust, firing ranges). So far, however, there is a paucity of studies investigating environmental factors contributing to ALS in veterans and even fewer assessing their exposure using biomarkers. Herein, we discuss ALS pathogenesis in relation to a series of persistent neurotoxicants (often emitted as mixtures) including: chemical elements, nanoparticles and lipophilic toxicants such as dioxins, polycyclic aromatic hydrocarbons and polychlorinated biphenyls. We propose these toxicants should be directly measured in veteran central nervous system tissue, where they may have accumulated for decades. Specific toxicants (or mixtures thereof) may accelerate ALS development following a multistep hypothesis or act synergistically with other service-linked exposures (e.g. head trauma/concussions). Such possibilities could explain the lower age of onset observed in veterans compared to civilians. Identifying high-risk exposures within vulnerable populations is key to understanding ALS etiopathogenesis and is urgently needed to act upon modifiable risk factors for military personnel who deserve enhanced protection during their years of service, not only for their short-term, but also long-term health.

## Introduction

Amyotrophic lateral sclerosis (ALS) is a predominantly sporadic, paralytic, and fatal condition caused by motor neuron degeneration [1]. Despite the identification of disease-causing mutations for ~ 10% of cases [2–4], the pathogenic mechanism(s) of ALS remain elusive, hampering the development of effective preventive or therapeutic strategies. It is thought there are likely as many types of toxic exposures leading to sporadic ALS as there are genetic variants causing familial or inherited ALS (> 25 causative genes) [4]. Interestingly, not all carriers of ALS-causing mutations develop ALS, even in old age. For instance, most mutations in TDP-43 exhibit a reduced penetrance [5]. There is also a large disparity at what age familial ALS presents. These observations suggest that, even in familial ALS, there are environmental influences. While sporadic ALS occasionally results from oligogenic causes [6, 7], it is generally believed that most sporadic cases result from a combination of environmental factors and genetic susceptibilities. Currently, identifying rare genetic susceptibility factors that synergistically interact with a given environmental exposure requires a large case–control analysis, and often through the efforts of a global consortium.

The importance of environmental exposures in ALS etiology is supported by reports of high discordance of this disease in monozygotic twins [8, 9], conjugal ALS [10] and an increased risk of ALS for specific occupations [11], activities [12] and exposures (e.g. metals [13, 14], pesticides [15]). Relevant to veterans affected with ALS, disease risk increases, sometimes two-fold, with deployment [16] or total years of deployment [17]. Beard et al*.* also found ALS was positively associated with exposure to herbicides, pesticides, certain metals and burning agents [17]. Another study called genes and environmental exposures in veterans (GENEVA) used a retrospective exposure assessment to evaluate  which industrial and occupational exposures, next to genetics, could drive the higher risk of ALS among military veterans [18]. They found that raters' confidence-modified exposure scores revealed potential higher exposure to hydrocarbon solvents, chlorinated solvents, and pesticides. Such evidence suggests that military personnel might be exposed to a variety of neurotoxicants and carcinogens whose adverse effects only emerge after sustained long-term exposure.

A recent study reported that the prevalence of El Escorial-defined ‘definite’ ALS cases is significantly higher among Air Force personnel, as well as among tactical operation officers and health care workers compared to other service branches and occupations [19]. These branch-specific differences are not reported by all studies [17, 20, 21]. Although a topic of debate, the suggested variation in rates among military branches may indicate that there are disproportionate exposures and/or factors experienced by certain military personnel. Despite such reports, there is no clear connection between a given environmental exposure and ALS veterans to date. One possible reason for this gap in understanding is that only a few studies (notably Fang et al*.,* 2010 [22]) have evaluated biomarkers of physiological exposure to specific neurotoxicants, such as lead (Pb), in this population.

In this review, we assert that to reliably characterize the physiological burden of exposure in veterans, it is important to evaluate the potentially causal chemicals in disease-relevant tissues, such as the central nervous system (CNS). Importantly, the causative environmental exposure(s) likely occurred years prior to disease onset (e.g. 10 + years). Thus, due to changes of residence, retirement, etc., a given ALS patient is likely no longer actively exposed to the causal toxicants at the time of diagnosis. While epidemiological exposure assessments are therefore challenging, the direct measurement of persistent neurotoxicants from subject tissue remains feasible and offers many advantages. For instance, toxic metals, such as Pb, accumulate in bones where they have a half-life of up to 30 years [23]. As a result of aging and decreased activity, metals like Pb can be mobilized from bones, thus internally extending potential exposures that may have initially occurred years ago. Similarly, the CNS is also known to accumulate such metals with a longer half-life than most soft organs [24] as well as lipophilic toxicants with stable chemical structures due to the high lipid content of CNS tissue [25]. Although this approach may not capture all toxicants that contribute to ALS (e.g. those that are not persistent), persistent and non-persistent toxicants are often co-emitted, and thus tracking the persistent ones presents a feasible mechanism for the identification of fundamental sources of exposure that will provide an overall more informed understanding of ALS environmental etiology as a whole.

Together, there are several questions that could greatly increase our understanding of the particular vulnerability of military veterans to ALS:

1. What toxic environmental exposures/toxicants are associated with ALS?
2. What concentrations and/or (sub)cellular accumulations of such toxicants are relevant to disease?
3. Are there synergistic patterns between associated toxicants and predisposing/lifestyle factors (e.g. prior head trauma, spinal cord injury, smoking, occupation etc.)?

## Similarities in disease development between ALS and cancer

In 2014, a groundbreaking epidemiological study by Al-Chalabi et al*.* described the development of ALS as a multistep process, similar to cancer [26]. The study was based on the premise that both ALS and cancer share characteristics such as the “onset being more common in later life, progression usually being rapid, the disease affecting a particular cell type, and showing complex inheritance” [26]. Using ALS registries from Ireland, the Netherlands, Italy, Scotland and England, Al-Chalabi and colleagues demonstrated a linear relationship between log incidence and log age in all five registries that was consistent with a six-step process [26], possibly derived from a combination of environmental exposures, genetic and other risk factors. Chiò et al*.* further demonstrated that patients with genetic mutations had fewer steps in their development of ALS, thereby supporting the idea of ALS as a multistep process [27]. Identifying exposures that serve as key steps or “hits” in ALS development will likley greatly increase our collective understanding of ALS etiology as well as the development of preventative and therapeutic strategies.

In this respect, military service members appear to represent a distinct subpopulation to which these principles could be applied. For instance, ALS incidence was found to be significantly higher during an approximate 10-year window following deployment for first Gulf War relative to those not deployed to the Gulf [28, 29]. Possibly following key exposures and/or interplay between toxicants and other risk factors, this defined window between service and disease onset provides a potential timeframe for the manifestation of ALS. Moreover, one study reports 85% of incident cases in Gulf War veterans within this window were < 45 years of age [30]. This decreased age of onset (typical ALS onset is ≥ 60 years in the general population) may also suggest that the exposures experienced by military personnel were more frequent and/or intense than that of persons developing ALS in the general population. We posit that service-linked exposures may represent one step (or several) in the proposed multistep process of ALS development [26].

## Military service and ALS

There are numerous reports suggesting an increased risk of ALS associated with military service [16, 31, 32]. A representative report by the United States National Academy of Medicine (US NAM, previously the Institute of Medicine) shows that, regardless of what war, ALS rates were generally increased and often doubled compared to rates in non-military individuals [16]. Although evidence based on such studies has often been deemed limited but suggestive (e.g. due to small samples sizes, given the rarity of disease), ALS is currently defined as a service-connected disease and the Department of Veterans Affairs (VA) has provided additional financial assistance to Gulf War veterans suffering from ALS [33]. As of 2010, more than 2 million service members have been deployed in support of Operation Enduring Freedom and Operation Iraqi Freedom [34]. ALS prevalence among US veterans deployed (2002–2015) was 19.7/100,000 [19]. Thus, understanding risk factors in the military setting could provide valuable insight and future mitigation strategies for current and future military service members.

The Gulf War serves as a notable case study when examining the connection between military service and ALS. In addition to a doubled incidence of disease compared to general population [28, 29], Haley observed that 8 years postwar, 85% of the incident cases in Gulf War veterans were < 45 years of age [30]. The observed incidence in young veterans increased from 1 to 5 cases per year and was not explained by “a change in the interval from onset to diagnosis or by a change in the US population death rate of ALS in those aged < 45 years” [30]. To this end, NAM have considered relationships between Gulf War illnesses in general (including ALS) and broad exposures to environmental toxicants including insect repellents, such as *N,N*-diethyl-m-toluamide (DEET), oil well combustion products such as organic hydrocarbons (VOCs), diesel exhaust, and various polycyclic aromatic hydrocarbons (PAHs), or other substances likely to be encountered during service such as acid aerosols, hydrazines, mustard agents, sarin, pyridostigmine bromide, and depleted uranium [35]. However, NAM found insufficient evidence to demonstrate that subclinical exposures to these agents was associated with specific patterns of illness [36]. Previous studies probing this connection have been criticized in part for (1) a lack of evidence of a biologically credible environmental exposure that could result in such an increase in neurodegenerative illness (no reliable biomarker of CNS exposure) and (2) the statistical methodologies studies used [37–41]. In regards to the latter, however, subsequent investigations have confirmed the increased risk of ALS among deployed personnel using capture/recapture methodologies [42].

Although the etiology of ALS in veterans remains unclear, it is clear military service represents a unique circumstance that aggregates a special group of collective factors. For instance, military service members are exposed to multiple environmental hazards during deployment (e.g. pollutants from unregulated industry, particulate matter from desert environments, exhaust from military vehicles and aircraft, emissions from open-air burn pits and toxicants on military bases); among the myriad of toxicants present in these service-connected exposures, several were linked to diseases (e.g. lead, pesticides, dioxins, and even aerosolized cyanobacteria pertaining to Gulf Wars [43]). Soldiers also receive prophylactic treatment of cholinergic inhibitors, which have been linked with neurodegenerative disease and potentially ALS [17, 44], to protect them against nerve gas and insect pests.  Beard et al*.* additionally identified numerous exposures associated with ALS and those with odds ratios > 3 included: treatment with nasopharyngeal radium, mixing or application of herbicides or burning agents as well as exposure to ground-level fumigation, nearby missile explosions, chemical agent-resistant compound paint or depleted uranium for munitions/armor [17]. Thus, while an effort has been made to link exposures to veteran service [32], no toxic exposures was demonstrated for its causal role in ALS.

In addition to environmental exposures, predisposing behavioral activities have also been considered in the context of military service and ALS. It has been postulated that the extreme physical exertion experienced by military personnel might also heighten ALS risk as well as smoking and alcohol consumption. However, variation in postwar ALS rates between military branches are not consistent with this idea. Altogether, previous attempts to establish a connection between military-linked environmental exposures and ALS based on historic records and questionnaires has proven difficult. Thus, evaluating the physiological dose of potential neurotoxicants in target tissues using quantitative and advanced molecular techniques would allow for stronger and improved correlations to be made between persistent toxicants and ALS. Although certain environmental toxicants have short half-lives and would not be measurable in postmortem CNS tissue, selecting toxicants that are (1) likely to contribute to disease based on evidence from the literature (see below) and (2) would be measurable as much as a decade or more after exposure will likely yield robust information. Potential toxicants include chemical elements, nanoparticles (NPs) and persistent, lipophilic toxicants such as dioxins, polycyclic aromatic hydrocarbons (PAHs) and polychlorinated biphenyls (PCBs). These environmental toxicants and their sources are described in more detail below.

## ALS-relevant and military-linked environmental exposure sources

### Diesel exhaust

Certain occupational studies have found increased ALS risk among persons in occupations with high exposure to diesel exhaust [45], such as truck drivers [46, 47] and construction workers [48], machine operators [49] and bus drivers [49]. Thus, diesel exhaust represents a proposed common denominator for ALS. Diesel engines of comparable power produce between 2 and 10 or up to 40-fold more particulate emissions than gasoline engines without or with a catalytic converter, respectively [50]. Moreover, inhaled diesel exhaust particles are ‘biopersistent’ and may carry additional chemical compounds on their surface [51]. Thus, the importance of diesel engine emissions to human health is not only a reflection of the toxic chemicals produced, but also the ability of particulate matter to ‘trap’ additional chemicals, thereby potentially extending the duration of exposure [51]. As a result, this may allow for the prolonged contact of passenger chemicals with the respiratory epithelium and increase their chance of uptake and subsequent initiation of oxidative stress, systemic inflammation and de novo mutations from resultant genomic damage [51, 52]. Diesel exhaust also contain PAHs such as benzo(a)pyrene and as well as additional toxicants and carcinogens (acetaldehyde, acrolein, benzene, 1,3-butadiene, ethyl benzene, formaldehyde, n-hexane, naphthalene, styrene, toluene, and xylene) identified by the US Environmental Protection Agency (EPA) [53]. Accordingly, one must consider diesel exhaust-derived toxicants as potential risk factors for neurodegeneration and, more specifically, ALS.

Living in close proximity to heavy traffic and air pollution is associated with a higher incidence of dementia [54] and risk of neurodegeneration. For example, fine particulate air pollution (particulate matter < 2.5 microns; PM_2.5_) has been linked to Alzheimer’s disease (AD) and Parkinson’s disease (PD) [55]. Specific to ALS, one recent study found suggestive evidence of increased ALS risk due to long term, high-concentration exposures to particulate matter < 10 microns (PM_10_) [56]. However, the statistical imprecision of the risk estimates due to the small sample size limited their capacity to definitively conclude an increase in risk [56]. In another study, Nunez et al*.* suggest that increased PM_2.5_ concentrations in New York state may contribute to the clinical aggravation of PD and ALS, especially for subjects > 70 years of age [57].

### Jet exhaust

Detrimental health effects have been observed for those working or residing in close proximity to jet emissions, including certain cancers [58, 59]. As evidenced in vivo*,* exposure of mice to particles collected at two separate airports via intratracheal installation induced pulmonary acute phase response, inflammation and genotoxicity [60]. In connection with ALS, the literature suggests that civilian airline flight attendants, pilots and navigators likewise have higher rates of this disease [59, 61]. Given that ALS prevalence has been found higher for Air Force service members relative to other military branches by certain studies [19, 29], we speculate that exposure to jet exhaust may serve as a contributing factor.

Products from jet emissions include ultrafine combustion particles, lubrication oils (including organophosphate esters), PAHs, volatile organic compounds and chemical elements including: Pb, Copper (Cu), Chromium (Cr), Nickel (Ni), Iron (Fe), Zinc (Zn), and Aluminum (Al) among others [58]. Although we are aware of their production, the toxicity of aircraft emissions has not been extensively researched. Emitted particles are generally ultrafine (< 100 nm). Like particles derived from other combustion sources, they may deposit in respiratory airways when inhaled and potentially cause irreversible damage to lung tissue, as observed for NPs generally [62, 63]. Researchers have investigated the toxicity of NPs from the exhaust of a CFM56-7B turbofan, the most common aircraft turbine engine. Using an aerosol deposition chamber, Jonsdottir et al*.* observed varying amounts cell death and oxidative stress in cultured bronchial epithelial cells depending on the combination of turbine thrust level and fuel type [64]. This is of importance as different thrust settings and fuels (including jet propellant (JP)-8 jet fuel) reportedly emit varying particle amounts [65]. Similar to the commercial jet A-1 fuel, JP-8 is a kerosene-based fuel that is conventionally used in Air Force aircraft, military vehicles and generators, thus, is common chemical exposure for Air Force-associated personnel (e.g. flight and ground crew) [65, 66]. Although no direct connection has been made, it is of note that Pugh et al. identified a significant increase in chronic obstructive pulmonary disease and asthma in veterans from the Iraq and Afghanistan Wars [67], which could possibly be associated with military-linked, environmental exposures. Exposure to jet fuel itself has been linked to hearing loss in both animal models [68] and humans [69]. Akin to urban pollution (described above), such auditory defects may also be potentially connected to NP-associated, brainstem pathologies [70].

Together, current evidence suggests that exposure to toxicants derived from diesel and jet engine exhaust can cause detrimental health effects. However, there are differences between the two sources. One study found the size distribution of aircraft exhaust particles, including lead dibromide, is smaller than that produced by automobiles burning the same leaded fuel (13 vs. 35 nm mean diameter for all particle types, respectively) [71]. In a separate study, although 10–1000 nm particles were associated with both roadways and aircraft, a principle component analysis conducted by the authors revealed features that differentiated the two sources: larger particle size and higher black carbon concentrations were a feature of roadways, while smaller particles and lower amounts of black carbon were associated with aircraft [72]. Such differences may be relevant to our understanding of emission-linked exposures when considering their contribution to disease development, although it is possible the overall physiochemical properties of each are ultimately similar [60]. Overall, the neurotoxic mechanisms and potential for such environmental pollutants to drive the increased ALS risk observed in military service members, airline workers and truck drivers needs further study.

### Brake and tire wear

In addition to the exhaust produced by combustion and other pollution sources associated with airports and air force bases, aircraft breaking and tire wear can also lead to the release of potential toxicants through the abrasion and subsequent production of smoke and ultrafine dust from these mechanical systems [65, 73]. For instance, experimental data collected at a major European airport have also reported high levels metals including barium (Ba), Zn, molybdenum (Mo), Cu and antimony (Sb) from runway smoke [74] and separate study also observed the production of trace elements generated by aircraft landing [75]. Although data are limited, one study estimates that the rubber lost from tires on varying aircraft can vary from tens of grams to ∼0.8 kg per landing [76].

### Burn pits

Burn pits are open areas for burning solid waste that were widely used in combat zones before 2009. A report by the Institute of Medicine described burn pits in Iraq and Afghanistan as burning waste that “consisted generally of 5–6% plastics, 6–7% wood, 3–4% miscellaneous noncombustibles, 1–2% metals, and 81–84% combustible materials” [77]. JP-8 was typically used as the accelerant and, although there are no official inventories, the refuse reportedly burned (i.e. plastics, metal cans, rubber, paints, solvents, petroleum, munitions and wood waste) produced hazardous emissions containing harmful particles and toxicants [77]. Unsurprisingly, exposure to burn pit emissions has been a cause for concern in relation to respiratory illness, cancer and neurological effects [77–79].

Pollutants investigated and detected during the evaluation of the Joint Base Balad burn pit included: dioxins, particulate matter (including metals), PAHs, volatile organic compounds [77]. Concentrations of polychlorinated dibenzo-p-dioxins and dibenzo-p-furans were overall low, but generally increased relative to urban environments. Additionally, a large contribution of the detected particulate matter, PAHs and volatile organic compounds were concluded to have likely originated from traffic and jet emissions as well as regional sources (e.g. normal human activity, dust storms, etc.). Thus, in combination with the presence of burn pits, such a nexus of exposures could potentially cause additive or synergetic health effects for those exposed. A public law mandated by Congress in 2013 required the VA to establish a registry for veterans with potential burn pit exposure in Iraq or Afghanistan through which participants can complete a questionnaire detailing their deployment/occupational, health and exposure history [80]. Together, these data could be used to epidemiologically investigate the link between burn pit exposure, in potential combination with other service-linked exposures, and ALS.

## Neurotoxic effects of toxicants

### PAHs

PAHs are by-products of combustion [81] and enter the human body from a variety of sources including: gasoline and diesel-fueled engines (e.g. jet fuel) [82], coal, solid waste, and oil burners [81], grilled and smoked meats [83], use of indoor fireplaces and stoves [84], and smoke from cigarettes [83, 85]. PAH residues are found frequently in suspended fine or ultrafine particulate matter in the air and inhalation is a major route of exposure [86]. They are lipophilic, are stored in fat tissues including those of the breast [87], and have been shown to cause mammary cancer in rodents [88]. In humans, autopsied samples revealed that the highest accumulation of PAHs occurred in abdominal fat and the brain [89]. At the toxicological level, some PAH compounds are able to bind the aryl hydrocarbon receptor (AHR), which regulates xenobiotic-metabolizing enzymes including some of the cytochrome P450s, ultimately resulting in increased DNA mutations [90, 91]. PAHs also have been found to be directly genotoxic [92]. In the rodent brain, a gene expression analysis showed that exposure to PAHs in the form of diesel exhaust emissions activates several genes associated with antioxidant defenses and inflammation [93]. In addition, benzo[a]pyrene (B[a]P), an individual PAH compound, has been identified for its neurotoxic potential [94]. An in vitro study elegantly demonstrated that B[a]P is not directly toxic to neurons; it kills neurons via a non-cell autonomous mechanism by activating microglia [95], the resident immune cells of the brain that have been  shown to play a key role in ALS [96]. Locomotor impairment after chronic exposure of B[a]P is significantly associated with development of neurodegenerative phenotypes typically affecting the dopaminergic system in zebrafish [97]. Histopathological observations in brain tissues showed a significant increase in pyknotic neuronal counts in the diencephalon and telencephalic region of zebrafish brain after B[a]P exposure and tyrosine hydroxylase, a marker of dopaminergic neurons, was reduced significantly in the exposure group. In addition to their neurotoxic effects, PAHs can be used to indicate exposure to incompletely combusted pollutants, such as soot. Soot particles can enter the brain and PAH compounds trapped inside the soot particles can be used to indicate the exposure from combustion [98].

### Persistent organic pollutants (POPs): PCBs and dioxins

PCBs and dioxins are known as persistent organic pollutants (POPs) and have all been found at military bases and in conjunction with service-linked activities as previously described. In general, POPs are resistant to environmental degradation and can bioaccumulate, resulting in adverse impacts on human health such as an increased risk of ALS [99] and reduced ALS survival in association with increased plasma concentrations [100]. PCBs and dioxins are lipophilic and accumulate in fatty tissues [101], including the brain. Interestingly, accumulation of PCBs in the brain was clearly shown not to have any regionalization [102]. However, in adolescent and neonatal male rats, exposure to PCBs was shown to induce brain region-dependent exacerbation (e.g., in the hypothalamus) or silencing (e.g., the prefrontal cortex) of genes implicated in neuroimmune function such as those coding for factors of the nuclear factor kappa b (NF-κB) complex [103, 104]. This suggests that despite homogeneous brain accumulation, PCBs can have brain area-specific neurotoxic effects probably based on regional gene–environment interaction. These two studies also indicate that neuroimmune dysregulation may be a prominent pathway of PCB neurotoxicity specifically in males. A similar sexual dimorphism is observed in ALS which preferentially affects men with a male:female ratio that lessens (from ~ 2.5 to 1.4) with age [105]. PCB exposure has been associated with reduced cognition in older adults [106] and the specific PCB, PCB-151, has an increased odds ratio in relation to ALS [107]. It is of note that POPs, such as PCBs [108], are associated with multiple neurodegenerative diseases, such as PD [109–118]. Although diseases like ALS, PD and AD do not share the same neuropathology, it is possible that an overlapping, genetic susceptibility occurs through pleiotropy and lends to the existence of common environmental triggers [119–121].

Dioxins are another class of POPs and are a component of certain pesticides, such as Agent Orange, to which ALS has been positively associated [17]. Moreover, elevated levels of the dioxin, 2,3,7,8-tetrachlorodibenzo-p-dioxin (TCDD), has been detected in breast milk from mothers living near the Vietnam Bien Hoa Air Base [122]. Exposure to TCDD from Agent Orange is believed to be an immunotoxin and the probable cause of several types of cancer in Vietnam veterans based on its clear carcinogenicity in experimental animal models [123]. One key mechanism proposed for TCDD carcinogenicity is epigenetic remodeling [124]. Supporting this view, exposure to dioxins or dioxin-like compounds has been associated with CNS developmental abnormalities in zebrafish and epigenetic modifications are well known to be particularly important during neuronal development [125]. Dioxins may also alter the expression of genes related to neuroimmune function [126], neurotransmission [127, 128], neurodevelopment [129, 130] and cytotoxicity [131, 132]. Overall, the molecular cascade by which the neurodegeneration-linked, chemical toxicants described thus far (dioxins, PCBs, and PAHs) contribute to disease is unclear, although one possibility is neuroimmune changes implicating in particular microglial cells potentially via the activation of the AHR. In vitro studies report agonists of the AHR, which interestingly was shown to induce a robust DNA demethylation of the Cyp1a1 promoter upon dioxin exposure [133], may induce up to a threefold increase in the ALS-linked TDP-43 protein in BE-M17 (human neuronal cell line), motor neuron differentiated iPSCs and the murine brain [134]. Although further research remains, such observations suggest the possibility that TDP-43 could be a potential molecular link between exposure to dioxins and ALS.

### Neurotoxic metals and elements

Specific metals have been linked to neurotoxicity and ALS [135–138]. In fact, exposure to certain metals was one of the earliest environmental risk factors proposed for ALS, although the jury is still out regarding their pathogenic role [139]. For instance, depending on the biospecimens used, there are discrepancies in the findings reported and peripheral measurements rarely reflect the CNS metal load [140, 141]. As described above, exposure to toxic metals is likely a major concern in veterans and a number of metals including Pb, mercury (Hg), selenium (Se), Cu, manganese (Mn), Fe and arsenic (As) certainly deserve further investigation in ALS patient, CNS tissue. These metals were selected for their relevance to ALS in the literature and our own epidemiological studies [12, 142–144] and are described in more detail below:

- **Lead (Pb)** was the first metal found to be elevated in cerebrospinal fluid (CSF), blood, and tibia from ALS patients [22, 145, 146]. Systematic reviews and meta-analyses cite Pb as an exposure with ‘convincing evidence’ of a causal link to ALS [147, 148]. Animal studies demonstrate the transport of Pb^2+^ ions across the blood–brain barrier [149] and the accumulation of insoluble TDP-43 in the cortex of exposed mice [150]. Based on the analysis of blood samples from occupationally exposed humans, there is evidence to suggest Pb-induced oxidative damage [151]. Relevant to military exposures, shooting at firing ranges results in the discharge of Pb dust, and elevated blood Pb levels that are associated with a variety of adverse health outcomes including essential tremor, cardiovascular morbidities and mortality, and decreased renal function [152]. In Denmark, occupational Pb exposure 10 years prior to diagnosis was associated with increased ALS risk (odds ratio 1.33, 95% confidence interval 1.03–1.72) [153]. Our own prior work linked activities involving Pb (e.g. casting lead bullets, making stained glass with lead joints and casting or using lead fishing sinkers) to ALS risk [144]. Moreover, Pb exposures 20 + years prior to diagnosis had larger effect sizes compared to those occurring more recently [144]. A further Australian study also linked petrol Pb emissions to ALS death rates both temporally and spatially [154].
- **Mercury (Hg)** is potentially a risk factor for ALS [142, 143]. This metal was elevated in the brain of seven ALS patients compared to controls [155] and is associated with increased risk in several studies [139, 147, 156]. Case reports of Hg poisoning have shown convincing ALS-like, clinical symptoms and support a causal relationship [157–159]. Our own regional and nationwide US studies demonstrated higher toenail Hg levels in ALS patients compared to controls [142, 143]. In mutant SOD1 ALS mice, Hg accumulates in spinal neurons [160] and rats exposed to methylmercury (2 mg/kg/day) exhibited ALS-like neurological effects [161]. In vitro proteomics studies reveal that methylmercury exposure causes electron transport chain dysfunction, oxidative stress and ubiquitin proteasome system impairment [135], pathological mechanisms all linked to ALS. Methylmercury neurotoxicity may also involve glutamate dyshomeostasis and excitotoxicity [162], an ALS-linked mechanisms that could be of particular relevance to the multi-stage hypothesis of ALS [26].
- **Selenium (Se)** was also linked to ALS [163–166]. Embedded shrapnel from explosive devices and retained bullet fragments also can increase serum levels of toxic metals including Se [167]. Higher risk of ALS was found in naturally seleniferous US regions [168, 169]. Veterinary and experimental animal evidence suggests that motor neurons are particularly vulnerable to Se [139, 164]. Despite Se being elevated in the spinal cord of ALS patients [163, 170], studies measuring Se levels in ALS-patient CSF and blood found both negative and positive correlations with ALS status [13, 166, 171]. Some of the discrepancies in these studies may be due to interactions among elements. For example, although not yet conclusively shown in humans, Se may counteract the absorption of methylmercury [172, 173].
- **Copper (Cu)** has often been a focus of ALS research due to its role as a cofactor of SOD1 [174–176]. Moreover, the pesticide, copper sulfate can dissolve in blood after exposure and be carried throughout the body as well as transported across the blood brain barrier (BBB) as a free Cu ion [177]. Increased Cu levels were measured in the spinal cord of mutant TDP-43 ALS mice [175] and in the blood of veterans with ALS [13]. However, in various peripheral biospecimens, trends in Cu levels were inconsistent [141].
- Excessive **manganese (Mn)** exposure is known to cause manganism, a neurological disorder resembling PD [178]. Moreover, Mn can cause ALS-like symptoms (such as muscle weakness) and ALS-like lesions of the corticospinal tract were reported in Mn-exposed workers [179]. In agreement, increased ALS rates have been reported in Mn miners and smelters worldwide [180, 181]. Elevated Mn levels were detected in the spinal cord of ALS patients and animal models [175, 182], whereas in patient body fluids, both negative and positive findings were reported [13, 174, 183, 184]. Roos et al. observed CSF Mn to be significantly increased compared to CSF of controls [184]. Notably, CSF Mn concentrations were higher than those in the plasma concentrations, suggesting transport of this element into the CNS.
- **Iron (Fe)** accumulation in the CNS has been clearly demonstrated in ALS patients, animal models and in vitro cell models [185–187]. In contrast, most studies using peripheral biospecimens did not find sizable differences in Fe levels in blood, hair, nail, CSF, and urine of ALS patients and controls [140]. Yet, Fe likely has a role in ALS as chelation of this metal is beneficial in a SOD1 ALS mouse model [188].
- Lastly, **arsenic (As)** exposure was shown to cause ALS-like motor neuropathy [189–191] and was suggested as a risk factor for ALS in association with folate deficiency [192]. A recent study reported lower As serum concentration in ALS patients, but As positively correlated with disease duration [136]. Prolonged As exposure in vitro triggers several features of ALS-linked TDP-43 pathology (e.g., mislocalization, aggregation) [193].

In addition to the ALS-linked elements described above, the following also possess evidence of an association with ALS: Using CSF (*n* = 17 cases, *n* = 10 controls), Roos et al. showed positive associations of ALS with Al, and cadmium (Cd), cobalt (Co), Zn, vanadium (V), and uranium (U) [137]. However, the Al finding was contradicted by a 1995 study of CSF that did not show elevation in ALS patients [185] as well as an additional study of temporal lobe tissue across neurological conditions did not find Al levels elevated in n = 16 ALS patients compared to age-matched controls [194]. Hozumi et al*.* found increased risk of ALS associated with Zn in the CSF (*n* = 52 cases, *n* = 15 controls). Zn was also elevated in CSF in a separate Greek cohort [195] as well as in the Roos 2013 CSF study [137], although this work was contradicted by Kapaki, who found no association with ALS and these elements in CSF (*n* = 28 cases, *n* = 36 controls) [196]. A further case study reported development of ALS in a battery worker exposed to high levels of Cd [197], although, elsewhere, Cd levels were lower in the CSF of ALS patients compared to controls [198]. Hozumi et al. found increased risk of ALS associated with elevated CSF magnesium (Mg) [183]. Using a cohort from the Danish National Patient Registry from 1982 to 2013 matched to controls, Dickerson et al. evaluated cumulative metal exposures estimated using job exposure matrices applied to occupational history. No statistically significant associations to ALS were discovered looking at Cr and Ni [199]. Lastly, based on studies of neurodegenerative illness in animals, Mo deficiency has been postulated to be a potential predisposing factor in ALS [200].

Several studies have directly assessed brain tissue for trace element levels. A review article of brain autopsy tissue sample measurements cites the detection of a large variety of the elements (see Table 3 of Grochowski et al. [201]), and provides quantitative reference levels for Cu, Zn, Mg, Fe, calcium (Ca), rubidium (Rb), Se, potassium (K), Mn, Al, silicon (Si), As, Ni, Pb, Cd, and Cr in various brain regions [201]. In studies of ALS, Cd and Zn were both significantly elevated in grey and white matter when Cd, Co, Cu, Fe, Mn, Rb, V, and Zn were measured in the brain tissue of *n* = 8 Guam ALS patients versus *n* = 5 controls [202]. By focusing on veterans, a population with increased ALS risk, and conducting a comprehensive assessment of elements in CNS tissue, one could potentially identify toxic elements that may not have been previously linked to ALS or examined in the general population. For example, uranium (U), Sb or tungsten (W; associated with munitions), rare earth and platinum group elements have not typically been measured in brain tissue.

The mechanisms of toxicity by which metals contribute to neurodegeneration in ALS remain to be clarified. However, in other neurodegenerative diseases like AD, multiple studies have validated that exposure to metals disrupts critical, immune-related pathways leading to chronic neuroinflammation and neuronal loss [203]. Metals have often been studied separately in terms of immunotoxicity and neurotoxicity, but one elegant study demonstrates that Pb, for instance, induces the production of autoantibodies against neural proteins, including myelin basic protein (MBP) and glial fibrillary acidic protein (GFAP) [204]. Therefore, Pb could contribute to the aggravation of neurodegenerative disease by exacerbating the immunogenicity of nervous system proteins. The consequences of metal dyshomeostasis in ALS has recently gained momentum and helped generate novel hypotheses, such as those linking mitochondrial dysfunction, intracellular calcium dyshomeostasis, pathological TDP-43 formation, pro-inflammatory microglia activation, and ultimately programmed neuronal cell death [205].

### Nanoparticles (NPs)

NPs are particles ≤ 100 nm in all dimensions and exist in a variety of shapes, sizes and compositions (organic, inorganic and carbon based). The nanometer scale of these particles allows for their direct interaction with cells and/or passage through biological barriers. Molecules (including proteins) can coat the NP surface, forming a NP corona [206, 207] and effectively become particle passengers [208]. Leveraging this property, NPs have been used as a therapeutic delivery mechanism for a variety of chemotherapeutic compounds [209]. However, NP pharmacological characteristics are often quickly altered in vivo as NPs become coated with biological milieu following delivery. The interaction of NPs with cells is influenced by the proteins and other molecules attached to their surface, as demonstrated by one ex vivo study reporting that the binding specificity of targeted NPs can be lost in the presence of plasma proteins [210]. The complexity and diversity of protein interactions with NPs to form the corona has not been completely elucidated [207]. While NP size and ability to transport molecules contributes to their therapeutic promise, these same characteristics may also interfere with vital cellular processes, resulting in cellular toxicity as well as human health and environmental concerns [63, 209, 211, 212]. For instance, silver (Ag) NPs have been predominately used for the development of medicines, drug delivery systems and medical device coatings as a result of their antibacterial properties [213, 214], yet multiple studies have demonstrated the toxicity of Ag NPs in vitro and in vivo [209]. Thus, the soft duality of NP features may also pose a detriment to a number of organs and highlights the need for a clearer understanding of these particles in biomedicine, manufacturing [215, 216] as well as general regulations for use [217].

As an environmental toxicant, NPs likely had a large impact in the days of Charcot when he first described ALS in the late 1860’s (e.g. as product of combustion). Today, additional sources of NPs posing a potential health hazard are found in the form of powders, suspensions, or sprays, which are universally used in textiles, paints, cosmetics, water disinfectants, food packing and ubiquitous in polluted environments with combustion emissions [218, 219]. Given these sources for exposure, the respiratory and gastrointestinal tracts, mucosa and even skin represent entry routes for environmental NPs [220–223]. While large aerosolized particles tend to remain in the respiratory tract, NPs may cross the respiratory epithelium to enter blood vessels [224]. Once in the bloodstream, select NPs can directly cross the BBB and/or damage BBB integrity and increase its permeability [225–228]. Additionally, numerous studies demonstrate that NPs can also bypass the BBB entirely via the olfactory system (nasal olfactory epithelial → olfactory bulb → brain) [222, 229–232]. Furthermore, the substantia nigra and brainstem have been suggested as targets for NPs via access the gastrointestinal tract and neuroenteric system [221, 222, 224]. Although our understanding of NP entry routes and subsequent toxicity is evolving, toxic NPs typically appear to be inorganic in nature and water insoluble; toxicity also appears linked to dose and frequency [233–235].

Once NPs reach the brain, they can access neurons, oligodendrocytes and glia they may alter the structure or activity of the nervous system and induce glial activation [236]. The primary neurotoxic mechanism of NPs is the generation of free radicals and induction of oxidative stress, which can damage biological macromolecules, inducing de novo mutations of DNA. In addition, NPs may directly or indirectly trigger apoptosis, autophagy, immune-responses, neuroinflammation and subsequent BBB damage [209, 237–247]. For instance, Xue et al*.* demonstrated SiO_2_-, TiO_2_-, and magnetite (Fe_3_O_4_)-NPs treatment caused microglial activation and cytokine secretion, resulting in PC12 toxicity and altered dopamine production [248]. Similarly, 10 and 30 nm Fe_3_O_4_-NPs were found to reduce dopamine rat brains as well [249]. Multiple factors may influence NP neurotoxicity including: size, shape, surface coatings, dissolution rates of metals, and interactions with specific cells and proteins [250]. Of particular, importance is size. In one study, Ag NPs (20 nm) exhibited increased cytotoxicity and pro-inflammatory response in cultured cells compared with larger particles (i.e. 80 nm) [251]. Similarly, a separate in vitro study indicated increased toxicity and oxidative stress from 20 vs 40 nm Ag NPs [252]. Ultimately, the exact neural damage and resultant neuropathology may depend on genetic susceptibility, individual NP characteristics and the differential access to target tissues achieved via their respective entry routes [253, 254] (Table 1).

**Table 1:** Examples of neurotoxic effects and mechanisms caused by environmental NPs

Nanoparticle	Mechanism and relevance to neurodegeneration
Iron oxides: magnetite (Fe_3_O_4_) iron oxide (Fe_2_O_3_),	Axonal transport and bypass the BBB via the nasal olfactory epithelium [222, 255, 256]
	Daily exposure affects synaptic transmission and nerve conduction, causing neural inflammation, apoptosis, induced neural antioxidant responses, and immune cell infiltration [257]
	Disrupted Fe homeostasis [258, 259], release of free Fe ions to catalyze the production of reactive oxygen species (ROS) through the Fenton reaction [249, 260] as well as the promotion of amyloid-β toxicity, as shown in vitro [261]
Silicon dioxide (SiO_2_)	Increased oxidative stress and altered microglial function; deleterious effects on the striatum and dopaminergic neurons [262]
	Intranasal administration in a mouse model lead to cognitive dysfunction and impairment, synaptic changes as well as pathologies similar to neurodegeneration [263]
	Induction of neuron depolarization in a cell culture model; no detected change in gene expression [264]
	PD-like behavioral changes in SiO_2_ NP-exposed Zebrafish model [265]
	Dose-dependent cytotoxicity and AD-like pathology in vitro [266]
Titanium oxide (TiO2)	Absorption and translocation into the brain by any portal of entry. Can further cross the placental barrier and accumulate in the fetal brain, causing impairments in the fetal brain development [267]
	Damage to BBB and induction of inflammatory response [268, 269]
	Exposure precipitates the development of neuropathological findings of early PD, AD and ALS, some of which appear to be manifested symptomatically [221, 253, 270, 271]
Nickel (Ni)	Ni NPs increased (Aβ)40 and Aβ42 levels in murine brains [272]

Given the need to better understand the neurotoxic potential of NPs, one important question is: what is the threshold of NP exposure for neurodegeneration or neurodegenerative pathology? In study of Mexico City subjects, an autopsy study showed hyperphosphorylated tau in the brainstem of an 11-month-old baby, who was found to have to 20 μg/m^3^ cumulative PM_2.5_ (calculated for age at death + pregnancy time), a fraction of the 2522 μg/m^3^ calculated for a 39 year old subject with AD neurofibrillary tangle advanced stages V-VI [270]. Calderón-Garcidueñas et al*.* additionally observed extensive structural organelle abnormalities in the substantia nigra involving mitochondria, endoplasmic reticulum and neuromelanin that were co-associated with the abundant presence of exogenous, Fe-, Al- and Ti-rich NPs in a population of young residents [221]. They also identified hyperphosphorylated tau, α-synuclein and TDP-43 in the brainstem of 182 Mexico City 27.29 ± 11.8y old Metropolitan Mexico City residents [221]. The co-existence of markers for two common neurodegenerative diseases (sporadic AD and PD), as well as the less common ALS/frontotemporal degeneration (FTD), suggests a common etiological denominator. Thus, NPs may act as catalysts for reactive oxygen species formation, altered cell signaling, protein misfolding, aggregation and fibril formation [221], hence, the co-clustering of such diseases (ALS, PD, dementia) in select geographical pockets [273]. Similarly, Fe-rich and TiO_2_-NPs may (even at low concentrations) may accelerate α-synuclein fibrillization [274], thereby representing a possible a pathomechanism that could potentially contribute to development of neurodegenerative-disease-linked pathology. Together, Calderón-Garcidueñas et al*.* have suggested that the properties of NPs, which result in cellular damage, potentially represent an additional pathomechanisms contributing to the development of neurodegeneration [221].

It is clear there is extensive and unregulated exposure to nanoparticles released in the environment and that emission sources are highly variable across military and civilian populations (Table 2). With the noteworthy progress of nanotechnology during the last decade, NP products will continue to be used increasingly in our everyday commercial products, industrial processes and medical applications. Thus, in addition to considering the courses of nanoparticle themselves_,_ other variables such as high traffic locations, residential areas, indoor environments, personal exposures, smoker, non-smoker, wind, season, etc. must also be taken into account to fully understand their potential risk.

**Table 2:** Veteran-relevant NP characteristics, Industrial Associations and/or Properties

Expected NP detection profile	Shape and size	Expected cellular/anatomical location and properties in vitro and in vivo
Elements from fuel combustion (gasoline, diesel, alternative mixed biofuels) and industrial sources (e-waste, lubricating oils) [275–282]: Fe, Pb, Ni, Cu, Cd, Hg, Al, Bismuth (Bi), Titanium (Ti)	Shape and size determine NP toxicity and capacity to reach target cells	Mitochondria, Golgi apparatus, lysosomes, phagosomes, and nuclei [221]
		Need to define localization of NPs in the neurovascular unit, including the BBB at endothelial level (integrity of tight junctions) [221, 289]
Technology-critical elements [283]:Gallium (Ga), Germanium (Ge), Indium (In), Tellurium (Te), Niobium (Nb), Tantalum (Ta), Thallium (Tl)		
Platinum Group Elements: Platinum (Pt), Palladium (Pd), Rhodium (Rh), Osmium (Os), Iridium (Ir), Ruthenium (Ru)		
Rare Earth Elements:Yttrium (Y), Lanthanum (La), Cerium (Ce), Praseodymium (Pr), Neodymium (Nd), Samarium (Sm), Europium (Eu), Gadolinium (Gd), Terbium (Tb), Dysprosium (Dy), Holmium (Ho), Erbium (Er), Ytterbium (Yb), Lutetium (Lu) [205, 282, 284–287]	Coexistence of multiple metal NPs alter the original toxicity of individual NP [285, 288]	
Detection of metalloid, Si [290], which may be of relevance in the veterans from the Gulf Wars where desert dust (e.g. SiO_2_) and particulate matter are prevalent [291]	Si NPs 7 + nm evoke oxidative stress and mitochondrial dysfunction [292, 293]	Within neurons, microglia, oligodendrocytes, astrocytes. Greater vulnerability of astrocytes expected [294]
		Subcellular accumulation in: Mitochondria, axons [295] and Autophagosomes [221, 296]
Detection of Ti nanorods (versus spherical shape) to determine its industrial origin	Ti nanorods are associated with e-waste, while spherical Ti NPs are associated with food sources [297]	Storage in autophagosomes [221]
		Membrane damage, cell cycle interference, reactive oxygen species formation [296]

## Conclusions

The pathogenic mechanisms of ALS remain elusive and has hampered the development of prevention strategies and effective therapeutics for this fatal disease. Although efforts have been made in the veteran population to understand the observed elevated ALS rates, no definitive factors have been implicated. Thus, measuring persistent toxicants of interest in ALS-patient CNS tissue and, particularly in evaluating potential pathogenic exposures in veteran cohorts, is warranted. Such a research approach could provide the basis for environmental exposure associations that are not unique to ALS, but potentially other neurodegenerative diseases with shared pleiotropy and to which veterans are also at higher risk [119–121, 298, 299]. To address this major challenge in ALS research, the field needs to further develop brain banks. In the traditional line of the Armed Forces Examiner System and Institute of Pathology [300], the Veterans Affairs Biorepository established a Brain Bank (VABBB) which provides a collection of carefully characterized, prepared and preserved CNS samples [301]. Moreover, linking these specimens to comprehensive demographic, lifestyle, residential, occupational, and clinical data are critical for the identification of novel associations between ALS and environmental toxicants. The Veterans Affairs Cooperative Studies Program Epidemiology Center in Durham, North Carolina (CSPEC-Durham) has assembled such a repository including extensive research data, genomic data, and study specimens (e.g., DNA, blood) for different content areas including ALS [302].

In summary, it will be crucial to evaluate service-linked toxicants such as PCBs, PAHs, dioxins, metals and NPs in veteran CNS tissue, none of which have been adequately evaluated in relation to neurodegenerative disease risk. These toxicant exposures in veterans appear to be persistent and cumulative, thereby, potentially allowing one to assess the link between ALS and past exposures. Based on the literature reviewed here, we hypothesize that the concentrations and/or distribution of proposed neurotoxicants will be increased and/or in the CNS tissue of ALS compared to controls. Advanced statistical techniques could be applied to clarify the ALS multistep hypothesis, evaluate toxicant synergy as well as anatomical and/or (sub)cellular locations. Finally, as brain banks often also collect genetic variant data, an evaluation of gene and environmental interactions could also be undertaken [121], thus enabling individualized risk assessments and exposure prevention strategies for susceptible individuals.

During their years of service, military personnel voluntarily expose themselves to short-term, life-threatening risks. Moreover, they may also often and unknowingly expose themselves to environmental factors as part of their duties, which can have dramatic consequences for their long-term health and lifespan. It is our responsibility as environmental health scientists to devise the best research strategies to clearly identify such factors, link them to disease and alert competent governmental agencies. The risk(s) associated with adverse exposures are certainly modifiable by prevention and mitigation strategies (e.g. personal protective equipment, exhaust emission control and reduction systems), which could be enabled by military authorities at lower costs than those associated with highly debilitating chronic diseases such as ALS (e.g., average total disease duration cost per patient for care and service in the U.S. is $1,433,992, excluding societal cost and family support cost [303]). We are hopeful that further research will address the urgent need to act upon modifiable risk factors for military personnel who deserve enhanced protection during their years of service for both their short- and long-term health.

## Data Availability

Not applicable.
